# Re: “A Case Report of Poor Response to Selpercatinib in the Presence of a 632_633 RET Deletion” by Wijewardene et al.

**DOI:** 10.1089/thy.2023.0258

**Published:** 2023-12-07

**Authors:** Sylwia Szymczak, Anna Szpurka, Zhao Hai Lu, Jack A. Dempsey

**Affiliations:** ^1^ Eli Lilly Polska, Department of Clinical Operations, Warsaw, Poland.; ^2^ Eli Lilly and Company, Indianapolis, Indiana, USA.

**Dear Editor:**

In this letter to the editor, we wish to provide additional information and clarification relevant to the article by Wijewardene et al. entitled “A case report of poor response to selpercatinib in the presence of a 632_633 RET deletion” published in *Thyroid* in January 2023.^1^

Experimental evidence and clinical observations from the LIBRETTO-001^2^ trial suggest that the *RET* p.632_633del mutation is neither rare in patients with medullary thyroid cancer (MTC) nor is it resistant to therapeutic intervention with the highly selective and potent REarranged during Transfection (RET)-inhibitor selpercatinib, a conclusion that is contrary to that presented in the mentioned case report. Findings by Elisei et al. show that such MTC-harboring somatic *RET* indels are common and can contribute to aggressive disease characteristics but respond to treatment with highly selective RET inhibitors.^3^

Among 319 MTC patients enrolled in the LIBRETTO-001 study, 9 MTC patients harbored p.632_633del RET mutation at exon 11 (c.1894_1899 del GAGCTG), all of whom derived clinical benefit from selpercatinib treatment. Baseline Eastern Cooperative Oncology Group for all 9 patients was 0–1 and all patients were treated at a dose of 160 mg selpercatinib twice a day (BID). Before receiving selpercatinib, 6 of the 9 patients were pretreated with the multikinase inhibitor vandetanib, achieving a best overall response of 1 partial response (PR), 3 stable disease (SD), and 2 progressive disease.

**FIG. 1.**
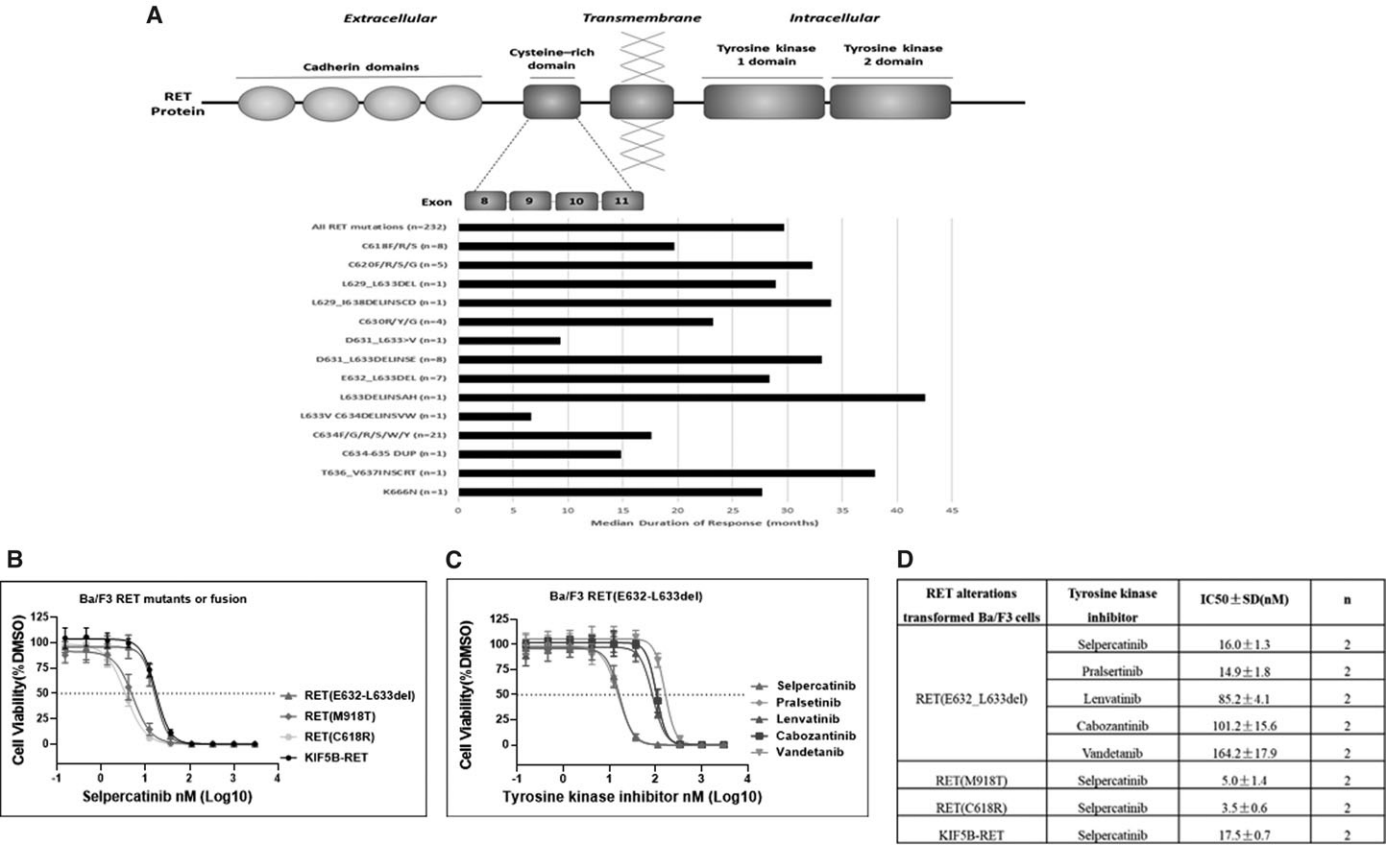
**(A)** Median DOR in months for all MTC RET mutants affecting the extracellular cysteine-rich domain (exons 8–11) from LIBRETTO-001. The median DOR for all cysteine-rich domain mutants is shown by black bars. Median DOR for RET (E632_L633 del) is comparable with the DOR of the overall MTC population and MTC patients with other mutations in the extracellular cysteine-rich domain. **(B)** Various RET mutants or RET fusion KIF5B-RET-transformed Ba/F3 cells were treated with selpercatinib. **(C)** RET (E632_L633 del)-transformed Ba/F3 cells were treated with tyrosine kinase inhibitors. **(D)** IC_50_ value summary. To assess the growth inhibitory effect of kinase inhibitors, transformed Ba/F3 cells were treated with various concentrations of compound in IL-3-free medium for 96 hours. Cell viability was measured by CellTiter-Glo assay at the end of treatment, and IC_50_ values were generated for each compound with Prism GraphPad. Data are presented as mean ± SD. DMSO, dimethyl sulfoxide; DOR, duration of response; IC_50_, half-maximal inhibitory concentration; IL-3, interleukin-3; MTC, medullary thyroid cancer; RET, REarranged during Transfection; SD, standard deviation.

After treatment with selpercatinib, 7 of the 9 patients had a PR (78%), and 2 maintained SD for at least 16 weeks (44 and 144 weeks, respectively). Median duration of response (DOR) was 28 months (4–42). This median DOR is comparable with the DOR observed in the overall MTC population (Retsevmo SmPC^4^), as well as in MTC patients with other mutations in extracellular cysteine-rich domain (CRD) of RET (Fig. 1A). The time on treatment of patients with p.632_633del RET mutation ranged from 12 to 50 months (median 38 months). Median progression-free survival was 31 months (10–45).

As of January 9, 2023, 6 patients were alive, while 4 continued to receive selpercatinib. The study received approval from ethics committee(s) in respective institutions and was conducted in accordance with the Declaration of Helsinki 174 of 1964, and all applicable country and local regulations. All patients provided written informed consent for study participation. Clinical outcomes of MTC patients with other mutations in the extracellular CRD of RET enrolled to the LIBRETTO-001 study suggest that this receptor with a 6-base pair in-frame deletion responds to RET kinase inhibition in a manner similar to cysteine substitutions and other deletions and insertions in the CRD.

These observations of clinical efficacy of selpercatinib in MTC patients with RET E632_L633del are supported by preclinical data from a functional assay on Ba/F3 cells transformed with RET (E632_L633del) or other relevant RET alterations. Results showed that interleukin-3 independent proliferation of RET (E632_L633del)-transformed Ba/F3 cells was potently inhibited by selpercatinib. The IC_50_ value for RET (E632_L633del) was consistently at ∼16 nM and was 4- to 5-fold higher than those for RET (M918T) and RET (C618R), but comparable with KIF5B-RET (Fig. 1A–C).

At concentrations >33 nM, selpercatinib completely abolished RET kinase-driven Ba/F3 cell proliferation regardless which *RET* mutant was assessed. At the clinical dose of 160 mg, concentration of selpercatinib is estimated to reach and maintain at >100 nM, which is sufficiently higher than the maximal inhibitory concentration for RET (E632_L633del) as determined by this cell-based assay. Results further showed that selpercatinib was more active against RET (E632_L633del) than multityrosine kinase inhibitors such as cabozantinib, vandetanib, or lenvatinib in this assay (Fig. 1C, D).

Based on this larger set of MTC patients harboring the p.632_633del RET mutation, we provide evidence that these patients received benefit from treatment with selpercatinib. Our results may dispute the proposed association between the p.632_633del RET mutation and poor selpercatinib response in the MTC patient presented in the report by Wijewardene et al.^1^
